# Supplementary material for: Decreased cerebrospinal fluid orexin levels not associated with clinical sleep disturbance in Parkinson’s disease: A retrospective study
Source: PLoS One. 2022 Dec 30;17(12):e0279747. doi: 10.1371/journal.pone.0279747 (PMC9803214; doi:10.1371/journal.pone.0279747)
Supplement: Attachment — Submitted filename: 20221211rebuttalletter.docx [file pone.0279747.s004.docx]

PONE-D-22-21585

Decreased cerebrospinal fluid orexin levels not associated with clinical sleep disturbance in Parkinson’s disease: A retrospective study

December 12, 2022

Claudio Liguori

Academic Editor

*PLOS ONE*

Dear Editor:

Thank you for reviewing our manuscript, titled “Decreased cerebrospinal fluid orexin levels not associated with clinical sleep disturbance in Parkinson’s disease: A retrospective study” (PONE-D-22-21585), and for providing constructive feedback.

With these changes to our revised manuscript, we hereby resubmit for a secondary evaluation. We look forward to hearing from you regarding our submission. We would be glad to respond to any further questions and comments that you may have.

Sincerely,

Kensuke Ikenaka and Hideki Mochizuki

Department of Neurology, Osaka University Graduate School of Medicine, Osaka, Japan.

Tel: +81-6-6879-3571

Fax: +81-6-6879-3579

E-mail: ikenaka@neurol.med.osaka-u.ac.jp, hmochizuki@neurol.med.osaka-u.ac.jp

**Response to Reviewer and Editor**

**#1 Journal Requirements**

We note that you have stated that you will provide repository information for your data at acceptance. Should your manuscript be accepted for publication, we will hold it until you provide the relevant accession numbers or DOIs necessary to access your data. If you wish to make changes to your Data Availability statement, please describe these changes in your cover letter and we will update your Data Availability statement to reflect the information you provide.

**Answer to #1**

Thank you for the notion. Our data is now available in the Dryad repository (https://datadryad.org/stash/share/IFCMVjwCZFzhhIQncweMKlKNH0pP4Fs1h3khfr0VAD0), as we described in the online submission system. The dataset will be available in the citation below after publication.

(Ogawa, Takuya et al. (2022), dataset_of_CSF_orexin_level_in_PD_with_clinical_assessment, Dryad, Dataset, <https://doi.org/10.5061/dryad.905qfttq0>)

**#2  Additional Editor Comments**

The Authors proposed an interesting study about CSF orexin levels in patients with PD. Several studies already proposed the same topic and Authors should better delineate the novelty of their research. The main criticism is the lack of pathological reduction of CSF orexin levels in patients than controls, although CSF orexin levels at the mere statistical analysis resulted lower. Authors should better discuss their results.

**Answer to #2**

We agree with your point regarding the lack of pathological reduction of CSF orexin levels in patients compared to controls, although CSF orexin levels were statistically lower. We already mentioned the novelty in terms of the large sample and various sufficient clinical evaluations. We also added a new sentence to the introduction section (**p. 6 Lines 105-107**) to clarify our motivation for this study. We also reconstructed the discussion section to describe the minimal pathological meaning of a decreased CSF orexin level in PD compared to controls (**p. 18, Lines 350-351**; **p. 18, Lines 355-357**; **p. 20 Lines 398-400**).

**(Section 1. Introduction) Page 6, lines 105-107:**

“Since previous studies had insufficient samples or used inconsistent methods of clinical assessment [10,11,13,14,16,18,19,21], the diagnostic or prognostic value of the CSF orexin level in PD lacks evidence. In this study, we measured orexin levels in the CSF samples obtained from patients with PD to clarify the role of orexin as a biomarker of PD, in the following particulars:...”

**(Section 2. Discussion > Orexin in PD compared to controls) Page 18, lines 348**

“Our study showed that CSF orexin levels in patients with PD were lower than those in the controls. However, the pathological relevance of CSF orexin levels needs to be considered. The normal range for CSF orexin level defined for the diagnosis of narcolepsy is as follows: over 200 pg/mL is normal, between 110 and 200 pg/mL is intermediate, and under 110 pg/mL is abnormal [34].”

**Page 18, lines 354-356**

“Although one study showed that CSF orexin levels in patients with PD decreased to an intermediate range [13], other studies demonstrated normal CSF orexin levels in these patients [11,12].”

**(Section 3. Discussion > Correlation of orexin with clinical characteristics) Page 20, lines 395-397**

“Our study showed no significant differences in CSF orexin levels between patients with PD with and those without DAs. Since our study was retrospective, it is possible that DA had already been discontinued owing to adverse effects. A previous study showed an improvement in EDS and an increase in CSF orexin levels after switching from PPX to pergolide [14]. In clinical situations, the effect of DA use on orexin levels seems to be small, but further studies are needed to focus on cases with adverse sleep events.

Taking all the results together, we believe that the decreased orexin level in patients with PD does not exert a substantial effect on the PD pathologies, at least at the relatively early stage that we have examined.“
